# A pilot clinical study of complex rhegmatogenous retinal detachment treatment via foldable capsular buckle scleral buckling

**DOI:** 10.1186/s12886-023-02913-5

**Published:** 2023-05-04

**Authors:** Cheng Li, Baike Zhang, Xiao Tan, Yong Jia, Lisha Guo, Chunlei Wang, Yaofan Xing, Qiaoyun Li, Xuemin Tian

**Affiliations:** 1 The 988th Hospital of People’s Liberation Army Joint Logistic Force, Zhengzhou, 450000 China; 2 State Key Laboratory of Ophthalmology, Zhongshan Ophthalmic Center, Sun Yat-sen University, Guangzhou, 510060 China; 3 Xinxiang Medical University, Xinxiang, 453000 China; 4 Shenzhen Aier Eye Hospital Affiliated to Jinan University, Shenzhen, 518000 China

**Keywords:** Foldable capsular buckle, Rhegmatogenous retinal detachment, Scleral buckling, Pars plana vitrectomy, Foldable capsular vitreous body

## Abstract

**Background:**

To evaluate the feasibility of and identify problems in treating complex rhegmatogenous retinal detachment using foldable capsular buckle scleral buckling.

**Methods:**

This prospective clinical study enrolled five patients with complex rhegmatogenous retinal detachment treated with foldable capsular buckle scleral buckling at the 988th Hospital of People’s Liberation Army Joint Logistic Force, China. During the 24-week follow-up period, the patients underwent measurements of their best-corrected visual acuity, slit-lamp examination, indirect ophthalmoscopy, and visual field testing. Additionally, B-ultrasound and fundus photography of the patients’ retinal reattachments helped evaluate the treatment’s post-surgery efficacy. We determined the safety of foldable capsular buckle scleral buckling based on infection, eye pain, diplopia, elevated intraocular pressure, and other postoperative severe complications.

**Results:**

All five patients’ complex rhegmatogenous retinal detachments were successfully treated and evaluated via B-ultrasound and fundus photography after surgery. Visual acuity was enhanced in four patients 24 weeks after surgery, while the remaining patients developed diplopia after surgery. No other complications were observed.

**Conclusion:**

This pilot study preliminarily determined that foldable capsular buckle scleral buckling is feasible for efficient and safe treatment of complex rhegmatogenous retinal detachment. These results support this surgery as a potential and novel alternative to current extraocular procedures for treating complex rhegmatogenous retinal detachment.

**Trial registration:**

The prospective observational clinical study protocol was approved by the Institutional Review Board and Ethics Committee and registered at the clinical research center in the 988th Hospital of People’s Liberation Army Joint Logistic Force, China (9,882,019,000).

## Background

Rhegmatogenous retinal detachment (RRD) is the most common form. The retinal separation is caused by fluid ingress from the vitreous cavity to the subretinal space through a retinal ‘break’[1, 2]. RRD is classified as simple or complex RRD according to the retinal detachment location, field, and if accompanied by other complications [3, 4]. In complex RRD, detachment is caused by a sizeable retinal tear, multiple retinal breaks, or posterior breaks and could have potential associations with vitreous hemorrhage, proliferative vitreoretinopathy (PVR), or other fundus diseases [3–5]. Currently, pars plana vitrectomy (PPV) is the most suitable surgery for complex RRD [3, 6–10]. However, it still has several disadvantages, including accelerated development of cataracts in phakic eyes, increased risk of PVR and epiretinal membranes, low oxygen distribution in the vitreous, and high-cost [11–17].

In a previous preliminary study, we tentatively used foldable capsular buckle (FCB) external buckling to treat simple RRD in five patients, and all retinas were reattached with few complications [18]. Compared to traditional external surgery, this surgery is less invasive, easier to perform, has fewer postoperative complications, and is less painful for patients. A multicenter clinical study investigating simple RRD treatment via FCB scleral buckling is currently underway. But whether the procedure is appropriate for complex RRD is not clear [18].

We speculate that FCB scleral buckling combined with other adjuvant therapies, such as retinal photocoagulation, transscleral cryotherapy, and vitreous gas injection, may be suitable for treating partially complex RRD for several reasons. First, because FCB scleral buckling can generate a larger buckling area on the sclera, treating RRD with extensive retinal detachment or multiple adjacent retinal breaks may be better. Second, compared to conventional scleral buckling, FCB may be more accessible to implant and place relatively posteriorly, and it have a larger pressure area, so the pressure is smoother and the retina is more tightly attached. This make the surgery more suitable for patients with RRD with retinal breaks in the posterior region of the eye. Third, the pressure ridge is fairly smooth because the FCB generates spherical pressure, which can reduce the effect of retinal surface tension and enhance retinal reattachment. Although our previous experiments aimed at treating simple RRD, successful retinal attachment and fewer postoperative complications led us to speculate that FCB scleral buckling could be a less invasive and more straightforward approach to treating some cases of complex RRD [18].

In this study, we tentatively used FCB to treat patients with complex RRD who refused PPV surgery for various reasons. This pilot clinical study aimed to evaluate the feasibility of using FCB scleral buckling to treat complex RRD, identify problems that may arise during treatment, and lay the foundation for further research on FCB scleral buckling.

## Methods

### Ethics approval

The prospective observational clinical study protocol was approved by the Institutional Review Board and Ethics Committee and registered in the clinical research center of the 988th Hospital of People’s Liberation Army Joint Logistic Force, China (9,882,019,000). It adhered strictly to the principles of the World Medical Association’s Declaration of Helsinki of the World Medical Association. All patients were provided with an explanation of the purpose and design of the study, and they were informed that FCB scleral buckling is still an off-label treatment for RRD. The patients provided written informed consent before participating in the study.

### Subjects

This was a prospective study, and the patient inclusion criteria were as follows: to start, the patients had to have retinal detachments caused by one giant retinal tear or multiple retinal breaks that subtended less than 6 mm in their longest dimension or had RRD associated with pseudophakia, high myopia, vitreous hemorrhage, PVR, or other fundus diseases. Some patients were not candidates for PPV surgery for various reasons. The exclusion criteria were patients who were allergic to silica, had severe systemic illness, were intolerant of surgery, or had previously received other surgical treatments, including PPV, scleral buckling, or pneumatic retinopexy.

### Foldable capsular vitreous body

**Fig. 1:**
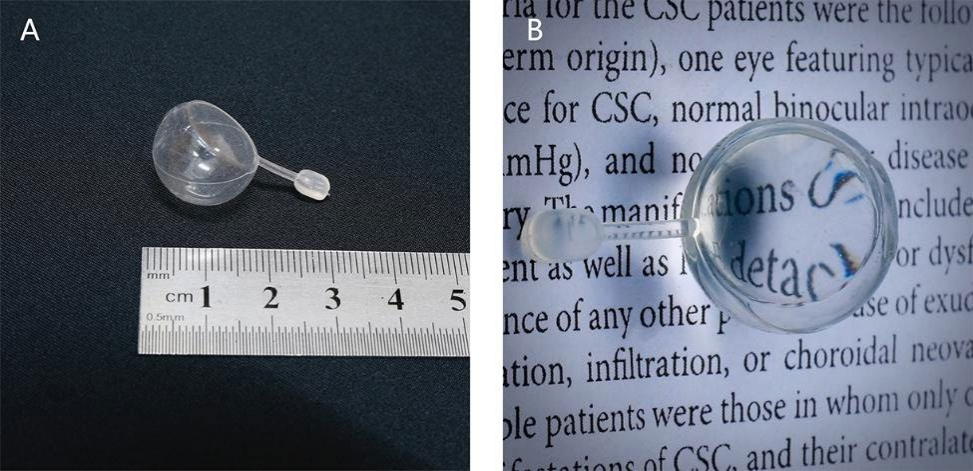
Foldable capsular vitreous body structure The FCVB was made of silicone rubber and consisted of a thin vitreous chamber–shaped capsule with a tube–valve system. A.The size and shape of the FCVB. B. FCVB is transparent after being filled with media

FCB is a commercially available product that is modified from foldable capsular vitreous bodies (FCVB) AV-10P model (Vesber, Guangzhou, China) [19]. It is made of silicone rubber and consists of a thin vitreous cavity–shaped capsule with a tube–valve system, as shown in Fig. 1. The BSS can be injected into the capsule through the tube valve system, which makes it ideal for this surgery [20].

### FCB scleral buckling and surgical procedure

All surgeries were performed by one senior ophthalmologist (Professor Xuemin Tian) at the 988th hospital, and we followed a previously published surgical method without implementing significant changes [18].

The surgery was conducted as follows: After subconjunctival infiltration anesthesia, the conjunctival sac was repeatedly washed with diluted iodophor and saline. The bulbar conjunctiva was cut 5 mm behind the limbus and parallel to the limbus along the direction of the retina tear. The incision was about 4 mm long, and the subconjunctival tissue was separated from the sclera. Along the scleral, backward separating the subconjunctival tissue to forming a tunnel of about 10 to 12 mm in length, and a radial incision of about 1 mm was made at the high point of the retinal detachment in the direction of the hole. A 25-G syringe needle was obliquely inserted into the subretinal space to release the subretinal fluid (about 0.6 ~ 1.0 mL), and then, the drainage hole was sutured. Under a microscope, the retinal holes were repositioned. The FCVB was vacuumed and folded, and the surgeon placed the FCVB along the premade tunnel with the spherical surface-facing sclera. The scleral incision was closed with a suture, and a 5.0 nonobservable suture was used to fixate the balloon drainage tube to 4-5 mm anterior to the posterior edge of the retina hole; then, 1 mL of normal saline was injected through the drainage valve device. We then checked the scleral pressure by the FCVB and further fixed all the drainage tubes. The balloon drainage valve was folded under the conjunctiva in a transverse direction, and the conjunctival incision was performed using two needles.

Tobramycin dexamethasone eye ointment was applied, and the affected eye was bandaged. During the operation, transscleral cryotherapy was applied conventionally, and conventional retinal photocoagulation was used postoperatively to close the hole and promote retinal reattachment. The observation was performed at least 12 weeks after surgery, and the balloon was removed after the laser treatment and when transscleral cryotherapy and photocoagulation spots appeared with obvious pigmentation. The procedure for taking out the FCVB this was performed in the operating room with subconjunctival infiltration anesthesia. However, some patients felt that their postoperative recovery was going well and that there were no apparent complications, so they were more willing to keep the FCVB for few more weeks.

### Pre- and post-operative observation

Before the operation, we conducted a routine ophthalmologic examination that included best-corrected visual acuity (BCVA) (LogMAR), intraocular pressure (IOP) (Canon TX-20, Canon Corporation, Japan; examiner: Lisha Guo), anterior segment examination with a slit-lamp, three-mirror contact lens examination and binocular indirect ophthalmoscope, fundus photography (TRC-50DX, Topcon, Japan; examiner: Qiaoyun Li), B-ultrasound examination (CineScan A/B, Quantel Medical, France; examiner: Lisha Guo), optical coherence tomography (OCT) (Spectralis, Heidelberg Engineering, Germany; examiner: Qiaoyun Li), eye axis measurement (CineScan A/B, Quantel Medical, France; examiner: Lisha Guo), and a general medical examination. A detailed fundus examination was also performed on the contralateral eye of the patients. Through the thorough fundus examination, we determined the scope of retinal detachment, either with or without the involvement of the macula, found the location of retinal tears, and determined the proliferative vitreoretinopathy grading.

After surgery, to evaluate the efficacy of FCB scleral buckling on retinal reattachment, we performed B-ultrasound, OCT, and fundus photography one week postoperatively (P1W). The primary postoperative outcome was the anatomical restoration of the retina at 24 weeks postoperatively (P24W). The postoperative observation included BCVA and recording of complications at one week, two weeks, three weeks, four weeks, 12 weeks, and 24 weeks post-surgery. The safety of the treatment was evaluated based on infection, eye pain, diplopia, elevated IOP, and other postoperative severe complications at 24 weeks after surgery.

## Results

All the clinical characteristics and surgical outcomes are summarized in Table 1.

**Table 1:** Clinical characteristics and surgical outcomes of the study subjects

Variables	Patient 1	Patient 2	Patient 3	Patient 4	Patient 5
Gender	Male	Female	Male	Male	Female
Age (years)	48	41	54	42	21
Onset of symptoms to the time of surgery (days)	10	2	3	4	30
Initial IOP (mmHg)	9	12	17	13	12
Eye axis (mm)	27.12	26.21	25.84	23.49	25.78
Other oculopathy	High myopia and posterior staphyloma	None	None	Pseudophakic eye	Proliferative vitreoretinopathy
Reason for refusing PPV	Surgery effect concern	Pregnant	Discomfort	Discomfort	Surgery effect concern
Initial BCVA	20/40	HM^*^	20/100	20/200	20/50
Hole location	Superotemporal	Superotemporal	Superotemporal	Temporal	Lower
Retinal involvement scope	2 quadrants	2 quadrants	2 quadrants	2 quadrants	2 quadrants
Macula affected	No	Yes	Yes	Yes	Yes
Intraoperative treatment	Condensation	None	Condensation	Condensation	Condensation
Post-operative treatment	Photocoagulation	Photocoagulation	Photocoagulation	Photocoagulation	Photocoagulation
BCVA at 1-week post-operation	20/40	20/80	20/80	20/50	20/50
BCVA at 24 weeks post-operation	20/25	20/40	20/32	20/32	20/40
Other adjuvant treatments	Daily injections of 250 ml 20% mannitol and 40 mg methylprednisolone sodium succinate	Intravitreal gas injection on tenth post-operative day	Released a small amount of fluid from the FCB	None	None
Discomfort and complications after surgery	Foreign body sensation and eye pain until the day after surgery	Subretinal fluid	Diplopia	None	None

^*^HM: Hand motion

Five patients were included in the study (three men and two women) with a mean age of 41.2 years (range: 21–52 years). The mean time from when the patients experienced a symptom of RRD to when they received surgery was 9.8 days (range: 2–30 days). The mean initial IOP was 12.6 mmHg (9–17 mmHg), and the mean eye axis was 25.688 mm (23.49–27.12 mm). The main reasons for refusing PPV included surgery effect concerns, meaning the patients were not satisfied with the effect of PPV on their contralateral eye, previous surgery, or they were encouraged by the better surgical outcomes of other subjects (2/5), pregnancy (1/5), or discomfort with PPV surgery (2/5).

### Evaluating the efficacy of FCB scleral buckling for complex RRD treatment

**Fig. 2:**
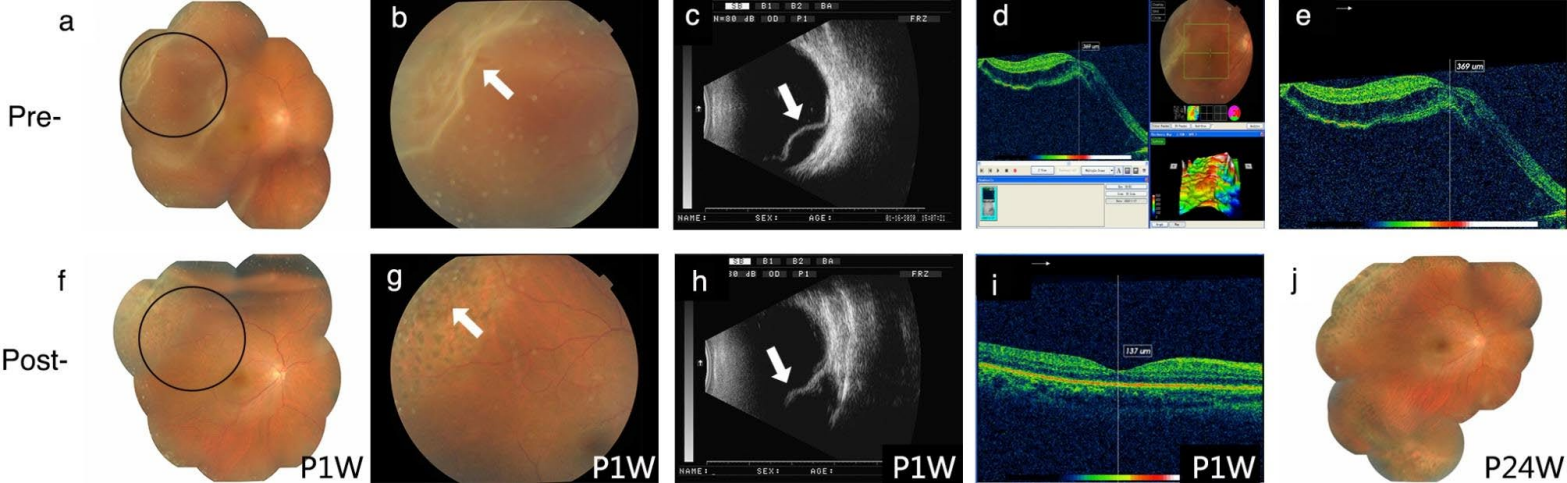
FCB scleral buckling efficacy on patient 1 **(a)** Fundus image showing retinal detachment pre-surgery. **(b)** The amplified circle area from **A** shows the retinal tear (white arrow). **(c)** B-ultrasound scan showing retinal detachment pre-surgery (white arrow). **d–e.** OCT shows an unaffected macula. **f.** Fundus image of FCB scleral buckling at 1-week post-surgery (P1W). **g.** Fundus image showing clear and distinct photocoagulation spots (white arrow). **h.** Post-operative B-ultrasound scan showing FCB’s evident pressure on the sclera (white arrow). **i.** OCT shows a small amount of remaining subretinal fluid and the mostly reattached retina. **j.** Fundus image at 24 weeks post-operation (P24W) showing the well-reattached retina

**Fig. 3:**
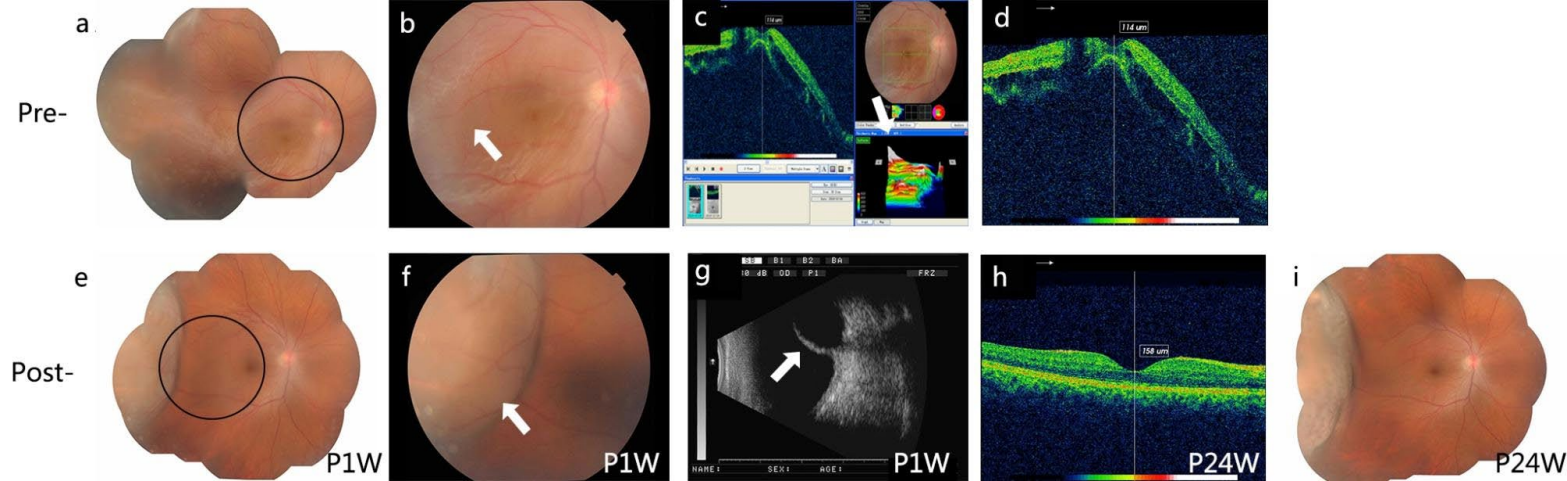
FCB scleral buckling efficacy on patient 2 **(a)** Fundus image showing retinal detachment pre-surgery. **(b)** The amplified circle area from **A** shows the retinal tear (white arrow). **(c)** Pre-operative B-ultrasound scan showing retinal detachment (white arrow). **d–e.** OCT shows an affected and detached macula. **f.** Fundus image of FCB scleral buckling at 1-week post-surgery (P1W). **g.** Fundus image showing clear and distinct photocoagulation spots (white arrow). **h.** Post-operative B-ultrasound scan results showing the FCB’s apparent pressure on the sclera (white arrow). **i.** OCT was showing the reattached macular. **j.** Fundus image at 24 weeks post-operation (P24W) showing the well-reattached retina

**Fig. 4:**
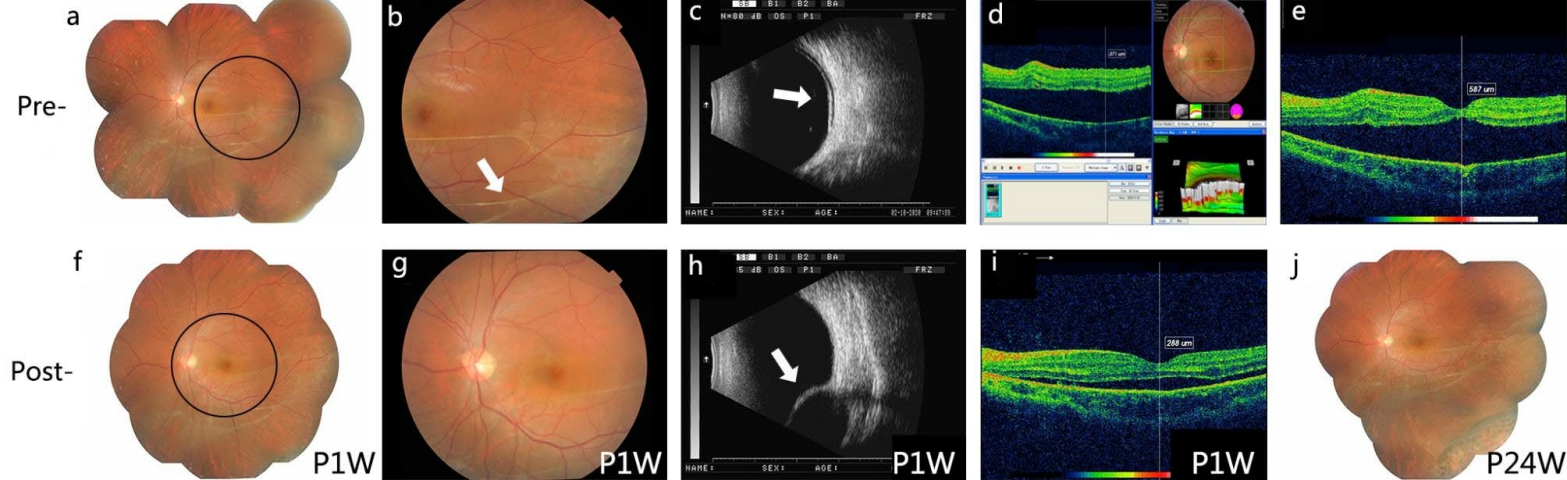
FCB scleral buckling efficacy with a giant retina tear in patient 3 **(a)** Fundus image showing retinal detachment pre-surgery. **(b)** OCT shows the affected macula and its unaffected central fovea. **(c)** Pre-surgery B-ultrasound scan showing retinal detachment (white arrow). **(d)** Fundus image of FCB scleral buckling one week post-surgery (P1W). **(e)** OCT shows no lesion present on the central fovea of the macula P1W. **(f)** B-ultrasound scan results P1W show the FCB’s apparent pressure on the sclera (white arrow). **(g)** Fundus image showing the retina attached at four weeks post-operation (P4W). **(h)** OCT shows a small lesion on the macula’s central fovea two weeks post-operation (P2W). **(i)** B-ultrasound showed better retinal restoration three weeks post-operation (P3W). **(j)** Fundus image showing the retina attached at 12 weeks post-operation (P12W). **k.** OCT showed a small lesion on the central fovea of the macula P3W. **l.** B-ultrasound scan showing the attached retina P4W. **m.** Fundus image showing the attached retina 16 weeks post-operation (P16W). **n.** OCT shows a small lesion on the macula’s central fovea at P12W. **o.** Fundus image showing attached retina 24 weeks post-operation (P24W).

**Fig. 5:**
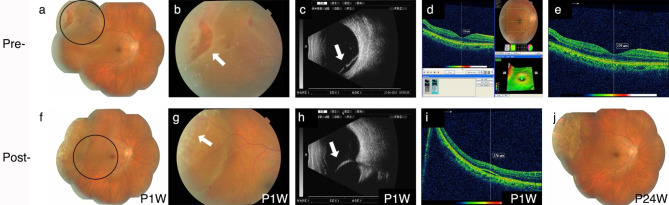
FCB scleral buckling efficacy in the pseudophakic eye of patient 4 **(a)** Fundus image showing retinal detachment pre-surgery. **(b)** The amplified circle area from **A** shows the detached retina (white arrow). **c**–**d.** OCT shows an affected and detached macula. **e.** Fundus image of FCB scleral buckling at 1-week post-surgery (P1W). **f.** Fundus image showing clear and distinct photocoagulation spots (white arrow). **g.** Post-operative B-ultrasound scan results showing the FCB’s apparent pressure on the sclera (white arrow). **h.** OCT was showing the reattached macular. **i.** Fundus image at 24 weeks post-operation (P24W) showing the well-reattached retina

**Fig. 6:**
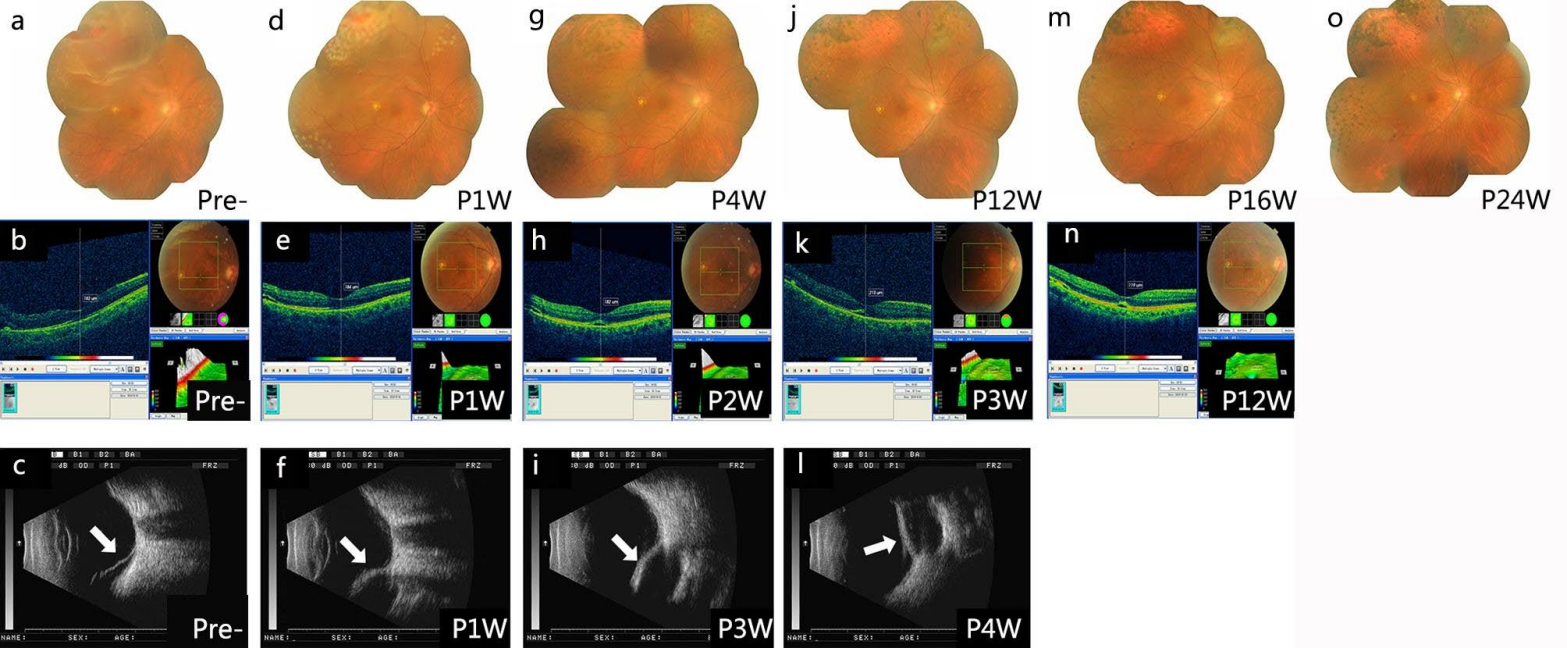
FCB scleral buckling efficacy in patient 5 **(a)** Fundus image showing retinal detachment pre-surgery. **(b)** The amplified circle area from **A** showing the proliferation cord (white arrow). **(c)** Pre-surgery B-ultrasound scan showing retinal detachment (white arrow). **d–e.** OCT shows an affected and detached macula. **f.** Fundus image of FCB scleral buckling at 1-week post-operation (P1W). **g.** Fundus image showing indistinct photocoagulation spots. **h.** Post-operative B-ultrasound scan results showing the FCB’s apparent pressure on the sclera (white arrow). **i.** OCT shows the much improved macular and a small amount of retained subretinal fluid. **j.** Fundus image at 24 weeks post-operation (P24W) showing the well-reattached retina

Three patients’ retinal holes were in the superotemporal region, one was in the temporal retina, and one was in the lower retina. The retinal detachment of all five patients involved two quadrants, and only one patient’s macula was unaffected. Four patients underwent intraoperative condensation, and all patients received postoperative photocoagulation. All patients had good retinal anatomical restoration at 24 weeks post-operation (P24W). At 1-week post-operation (P1W), three patients had enhanced BCVA; at P24W, all patients had improved BCVA. The retinal restorations of all patients are shown in Figs. 2, 3, 4, 5 and 6.

### Safety evaluation of FCB scleral buckling

Regarding safety, there were no adverse events during the 24-week postoperative follow-up, including endophthalmitis, cardiovascular events, acute high IOP events, and other systemic reactions. None of the patients had episodes of severe postoperative bleeding or unbearable discomfort, as observed in the safety evaluation in a previous study [18]. However, Patient 3 was found to develop diplopia and eye movement limitation the day after surgery until the FCB was removed after P4W. After the FCB was removed, the retina was still attached well. Additionally, patient 1 had foreign body sensation and eye pain, which spontaneously remitted on the second-day post-surgery.

## Discussion

This pilot clinical study preliminarily demonstrated that FCB scleral buckling could be an alternative treatment for retinal reattachment in some complex RRD patients who refuse PPV surgery. Considering a previous exploratory clinical study that used this technique to treat simple RRD, we believe that FCB scleral buckling may represent an innovative surgical method that can improve general RRD management [18].

From the postoperative examination results of five patients, it is clear that external FCB exerted pressure on the sclera and retina and that its location was not easily changed by ocular movement during the postoperative observation period. Combined with the subretinal fluid release, transscleral cryotherapy, and retinal photocoagulation, FCB scleral buckling surgery significantly improved the BCVA of the five patients compared to that before surgery, indicating that this innovative surgical method supports retinal reattachment and restores function. The retina was successfully reattached after surgery in all patients.

For patient 2, postoperative examination showed a small amount of subretinal fluid in the upper peripheral fundus; therefore, we administered an intravitreal gas injection on the tenth postoperative day to promote close retina attachment. The retina was fully reattached on the second day after the intravitreal gas injection. Postoperative OCT of patient 5 showed that the neuroepithelial retina was still slightly detached, possibly because of the patient’s long retinal detachment, viscous subretinal fluid, and severe PVR proliferation cord. However, a common scenario in patients undergoing retinal reattachment is slow absorption of viscous subretinal fluid and complete reattachment. Considering the severe retinal detachment in all five patients, combined with the particular case of high myopia, intraocular lens, or PVR, we showed that FCB scleral buckling is effective in treating complex RRD.

None of the five patients experienced severe adverse events, such as endophthalmitis, cardiovascular events, or other systemic reactions. Moreover, no acute high IOP events caused by FCB scleral buckling, severe postoperative bleeding, or extreme discomfort were observed. However, it should be noted that Patient 3 was found to develop diplopia the day after the operation, which did not resolve after the fluid was released from the FCB, but continued until the FCB was removed after P4W. This may have occurred because the FCVB influenced the extraocular muscles, especially since it was located under the rectus in accordance with the patient’s retinal detachment and hole location. However, the complication was resolved by removing the FCVB, indicating that its influence on the extraocular muscles was reversible. Because the FCB balloon is not continuously implanted under the conjunctiva, diplopia may occur in some patients; however, with the removal of the balloon, this issue is expected to resolve. Therefore, with these few complications and using the 3/n rule, we are confident that the actual complication rate of FCB scleral buckling is no more than 60% [21]. It should also be noted that in this study, we did not observe the blood supply of the anterior segment of eyes undergoing surgery, which may be an important impact of external surgery on eye health. Because recent studies have shown that one month after receiving scleral buckle, a uniform reduction of the iris vessel network developed[22]. Future studies need to further evaluate the effect of FCB scleral buckling on the blood supply of the anterior segment.

FCB scleral buckling is an improvement of traditional external scleral surgery because it has advantages and can avoid shortcomings. In external scleral surgery, the fundus image is inverted through an indirect ophthalmoscope, and the magnification is slight, making it difficult to perform and to be mastered. In addition, post-eyeball anesthesia is required to increase the risk of puncture. Moreover, the sclera must be exposed by repeatedly pulling the muscle, thus inducing pain in the patient and introducing a high risk of triggering the oculocardiac reflex. In contrast, in FCB scleral buckling, all these procedures are conducted under a microscope and involve fewer muscles, making it less invasive and easier to perform. All patients in this study received local surface infiltration anesthesia, which may indicate that this surgery is especially suitable for the elderly, young children, nervous patients, patients in poor health, and even pregnant women.

This type of surgery also has a more extensive pressure range, usually covering the hole and surrounding detached retina. Although the spherical pressure generated by the FCVB and its indwelling time can be controlled, we tentatively used FCB scleral buckling to treat a few patients with complex RRD. However, these patients should have received PPV surgery but refused. As a type of external scleral surgery, FCB scleral buckling has the common advantages of external scleral surgery. It could avoid some disadvantages of PPV surgery, such as the increased risk of PVR and epiretinal membranes and low oxygen distribution in the vitreous, which could further accelerate cataracts in phakic eyes and damage the trabecular meshwork cells [11–16]. FCB scleral buckling could also prevent optic nerve atrophy, disruption of ciliary body secretions induced by silicone oil injection, and other complications caused by PPV surgery [13, 23–30].

This study showed that FCB scleral buckling could be a feasible, effective, and safe alternative to retinal reattachment in complex RRD patients who refuse PPV surgery. However, certain shortcomings of this surgery should not be neglected, as it is relatively difficult to fix if the FCVB is located under the muscle. Patients with an FCVB risk developing temporary diplopia, although this can be relieved by releasing water from the balloon. Additionally, the current study design has some limitations, such as its small number of subjects, lack of a control group, and absence of a comparison with conventional treatment strategies. Accordingly, a large-scale randomized study controlled for PPV should be conducted further to confirm the efficacy and safety of FCB scleral buckling.

This study showed that FCB scleral buckling is a simple, easy-to-learn, less invasive, and less complicated alternative for treating some complex RRD patients who refuse PPV surgery. These promising results give us the confidence to conduct large-scale multicenter clinical trials further to verify the efficacy and safety of FCB scleral buckling.

## Data Availability

The datasets used and analysed during the current study are available from the corresponding author on reasonable request.

## Abbreviations

RRD: Rhegmatogenous retinal detachment
PVR: proliferative vitreoretinopathy
PPV: pars plana vitrectomy
FCB: foldable capsular buckle
FCVB: foldable capsular vitreous bodies
P1W: 1 week postoperatively
P24W: 24 weeks postoperatively
BCVA: best-corrected visual acuity
IOP: intraocular pressure
